# A Comprehensive Review of Minimally Invasive Dermatosurgical Procedures

**DOI:** 10.7759/cureus.56152

**Published:** 2024-03-14

**Authors:** Soham Meghe, Raavi Ramapure, Sharwari Jaiswal, Sugat Jawade, Sudhir Singh

**Affiliations:** 1 Dermatology, Jawaharlal Nehru Medical College, Datta Meghe Institute of Higher Education & Research, Wardha, IND

**Keywords:** innovations, clinical outcomes, surgical techniques, cosmetic dermatology, dermatologic procedures, minimally invasive dermatosurgery

## Abstract

Minimally invasive dermatosurgical procedures have revolutionized the field of dermatology, offering patients effective treatment options with reduced risks and downtime. This review provides a comprehensive overview of these procedures, beginning with their definition and historical context. We classify minimally invasive techniques, including both surgical and nonsurgical approaches, and explore their wide-ranging applications in cosmetic and therapeutic dermatology. Patient selection, preoperative assessment, techniques, clinical outcomes, and comparisons with traditional surgical methods are thoroughly examined. The implications for clinical practice are discussed, emphasizing the importance of integrating minimally invasive techniques into dermatologic care to enhance patient outcomes. Furthermore, areas for future research are identified, highlighting the need for ongoing studies to optimize techniques, evaluate long-term outcomes, and explore emerging technologies. Overall, this review underscores the significance of minimally invasive dermatosurgical procedures in advancing dermatologic practice and improving patient care.

## Introduction and background

Minimally invasive dermatosurgical procedures denote surgical methods executed with minimal disturbance to surrounding tissues, typically through small incisions or entry points. These procedures are geared toward achieving therapeutic or cosmetic goals while minimizing patient trauma, scarring, and recovery time [1]. The importance of minimally invasive dermatosurgical procedures in dermatology has grown due to their capacity to address various skin conditions and aesthetic concerns with minimal risk and downtime. These techniques offer patients alternatives to traditional surgical methods, resulting in quicker recovery, reduced discomfort, and improved cosmetic outcomes [2]. Advancements in technology and techniques have broadened the scope of minimally invasive procedures in dermatologic practice, providing solutions for an array of dermatologic conditions, including benign and malignant lesions and cosmetic issues like wrinkles, scars, and pigmentation problems [3,4].

This review presents a comprehensive overview of minimally invasive dermatosurgical procedures by examining their historical background, classification, indications, techniques, outcomes, and future directions. Its objective is to illuminate the significance of these procedures in modern dermatologic practice. Furthermore, the review aims to underscore the evolving role of minimally invasive techniques in meeting patients’ diverse needs, advancing the dermatosurgery field, and shaping the future of dermatologic care.

## Review

Historical perspective

Evolution of Dermatosurgery

The progression of dermatosurgery is a pivotal facet of the historical narrative of dermatology. Before the mid-20th century, dermatology predominantly centered around clinical practices with minimal surgical interventions. However, the landscape shifted notably in the 1950s and 1960s as dermatologists started integrating surgical procedures into their repertoire. This evolution birthed the development of techniques, publications, and technological innovations, ushering dermatology into a clinical-surgical specialty. This transition facilitated the widespread adoption of various dermatological procedures, catering to therapeutic and aesthetic objectives. Cryosurgery, a method employing freezing agents like liquid nitrogen to address skin lesions, has emerged as a widely utilized approach for treating benign and malignant dermatologic conditions [5]. Furthermore, advancements in dermatological surgery broadened the scope of practice to encompass an extensive array of surgical, diagnostic, and aesthetic procedures [4]. The historical journey of dermatologic surgery traces back to ancient civilizations, where diverse cultures contributed to the evolution of medical practices. The refinement of tools for minimally invasive surgery has been a gradual and ongoing process throughout history. In the 20th century, scientific breakthroughs and technological revolutions catalyzed further transformations in dermatological practices, introducing new therapeutic modalities and refining surgical techniques. The advent of minimally invasive cosmetic procedures is a testament to this evolution, offering patients less invasive options for skin rejuvenation and treatment [5-7].

Milestones in Minimally Invasive Techniques

Minimally invasive surgery boasts a rich historical lineage that dates back to ancient civilizations. The earliest documented instance of such procedures traces back to approximately 400 B.C. when Hippocrates employed a rectal speculum to examine and treat hemorrhoids. Across the centuries, there were steady advancements in tools and techniques punctuated by significant milestones. One milestone occurred in 1806 when Philipp Bozzini devised a “light transmitter,” laying the groundwork for illuminated endoscopy. Fast forward to 1982, when the advent of the first solid-state camera for laparoscopy revolutionized minimally invasive surgery. However, it was not until 1987 that Philippe Mouret’s pioneering performance of the first laparoscopic cholecystectomy truly heralded the dawn of the laparoscopic era, sparking a paradigm shift in surgical approaches [8]. The landscape of innovative minimally invasive surgery continues to evolve, with a trajectory focused on novel forms of image-guided therapy and the targeted delivery of treatments [9]. In dermatosurgery, minimally invasive cosmetic procedures have become commonplace for addressing many skin concerns. Techniques such as chemical peels, intradermal fillers, botulinum toxin injections, and microneedling are widely utilized to enhance and rejuvenate the skin [7].

Classification of minimally invasive dermatosurgical procedures

Surgical Techniques

Curettage and electrodesiccation: Curettage and electrodesiccation represent a dermatosurgical technique utilized in managing superficial basal cell and squamous cell carcinomas, along with certain precancerous skin lesions [10]. This procedure commences with the administration of local anesthesia to ensure patient comfort. Subsequently, the targeted skin tumor is excised using a slender curette with a sharp looped edge. Following excision, the area undergoes treatment with an electric needle (electrode), effectively eradicating any residual cancerous cells. This process may be repeated once or twice within the same office visit [10]. Often deemed suitable for noncritical regions like the trunk and extremities, where resultant scars are less conspicuous, curettage and electrodesiccation also find utility in the surgical removal of benign skin lesions, precancerous growths, and diminutive skin malignancies such as basal cell and squamous cell carcinomas [10,11]. Evidencing a commendable cure rate, this procedure is lauded for its efficiency and cost-effectiveness in addressing skin cancer [11]. Nonetheless, diligent post-treatment surveillance is imperative for cancerous lesions to avert the risk of recurrence, as residual abnormal cells may persist [11].

Cryosurgery: Cryosurgery, also known as cryotherapy, emerges as a minimally invasive intervention leveraging profoundly cold temperatures to eliminate aberrant tissue, predominantly tumors or precancerous lesions. Its utility extends to treating skin cancer, skin lesions, and select internal cancers like those affecting the prostate, cervix, and liver [12,13]. Employing liquid nitrogen or alternative cryogens, cryosurgery induces tissue destruction through freezing, facilitating subsequent cell demise and absorption by the body. The procedure may be conducted externally on the skin or internally via a cryoprobe introduced through a small incision [12]. The advantages of cryosurgery encompass minimal discomfort, diminished blood loss relative to conventional surgery, and minimal collateral damage to surrounding healthy tissue [14].

Nevertheless, potential adverse effects include blistering, nerve injury, infection, and scarring [12,13]. Preparation for cryosurgery varies based on the treatment site; skin cancer treatment typically necessitates no specific preparatory measures, while internal cryosurgery may entail discontinuing certain medications, such as aspirin or anticoagulants, before the procedure [12]. Following cryosurgery, patients may experience mild discomfort, erythema, swelling, and blister formation at the treatment site. Over-the-counter analgesics suffice for pain management, with healing times contingent on treatment location [15]. Multiple sessions may be necessary to eradicate abnormal tissue completely [15]. Ongoing research endeavors to explore the efficacy of cryosurgery across diverse cancer types, including oral, breast, and pancreatic malignancies [14].

Laser surgery: Laser surgery represents a surgical modality harnessing laser beams to incise, coagulate, vaporize, or ablate undesirable tissue. By directing a focused laser beam onto tissue, the energy is absorbed by chromophores within the skin, inducing tissue coagulation and necrosis, thereby facilitating the removal of targeted tissue. Various surgical laser types include carbon dioxide, argon, Nd:YAG, and potassium titanyl phosphate lasers. Laser surgery finds application across a broad spectrum of medical specialties, encompassing dermatology, plastic surgery, ophthalmology (e.g., LASIK), endovascular surgery, and foot and ankle surgery [16-19]. Renowned for its precision and ability to minimize collateral damage to surrounding tissues, laser surgery stands as an invaluable asset in modern surgical practice.

Radiofrequency surgery: Radiofrequency surgery, referred to as radiofrequency ablation (RFA), epitomizes a minimally invasive technique leveraging heat to obliterate tissue. It finds primary utility in managing various conditions, ranging from benign and malignant tumors to chronic venous insufficiency in the lower extremities, chronic back and neck pain, and joint pain affecting the knees, sacroiliac joints, hips, or shoulders [20-23]. A needle-like probe is introduced into the body during the procedure, emitting radiofrequency waves into the adjacent tissue, prompting cellular demise. Subsequent removal of these cells by the immune system typically leads to nodule shrinkage or pain alleviation [20]. Comparable to a needle biopsy, the procedure can be conducted in an office or outpatient setting, typically without general anesthesia. Following RFA, most individuals can return home on the same day as treatment and resume normal activities within a few days [20,22]. While generally well tolerated, potential transient side effects may include weakness, numbness, swelling, or bruising at the insertion site. In rare instances, permanent nerve damage or persistent pain may ensue [20,22].

Nonsurgical Techniques

Chemical peels: Chemical peels represent dermatologic procedures employing chemical solutions to exfoliate and rejuvenate the skin by removing its top layers. Chemical peels unveil smoother, more youthful skin underneath by shedding damaged skin cells, effectively addressing concerns such as wrinkles, acne, scars, uneven skin tone, and other imperfections. Various chemical solutions are utilized, including glycolic acid, trichloroacetic acid, salicylic acid, lactic acid, or carbolic acid (phenol), each penetrating to different depths within the skin. Selecting a chemical peel hinges on the desired outcome and targeted skin condition [24,25]. Chemical peels are categorized based on their depth into light, medium, or deep peels. Light peels typically entail minimal downtime, often featuring glycolic or salicylic acid. Medium peels delve deeper into the skin, potentially inducing redness and swelling for several days. Deep peels, frequently employing carbolic acid (phenol), yield more significant results but necessitate an extended recovery period [24]. Before undergoing a chemical peel, meticulous skin preparation is imperative, involving avoiding activities such as waxing and dermabrasion, sun limitation, and adherence to specific skincare regimens to optimize peel efficacy and mitigate complications like hyperpigmentation and scarring [25]. The procedure entails the application of the chemical solution to the skin, possibly eliciting a warm or stinging sensation. Post-peel, the skin may exhibit redness, tightness, irritation, or swelling. Following treatment, patients typically receive instructions encompassing sun protection, cleansing, moisturizing, and refraining from picking or scratching the treated skin. While makeup can typically be applied shortly after light peels, a longer waiting period may be warranted following medium or deep peels [24]. Consulting with a board-certified dermatologist or plastic surgeon is essential before undergoing a chemical peel to determine the most suitable type based on individual skin concerns and goals. Thorough patient evaluation, preparation, and post-treatment care are indispensable for achieving optimal outcomes with chemical peels [26,27].

Microdermabrasion: Microdermabrasion stands as a minimally invasive cosmetic procedure leveraging specialized instruments to gently exfoliate the skin’s outermost layer, thereby enhancing skin tone and texture while addressing issues such as fine lines, wrinkles, age spots, and uneven skin tone [28-32]. Primary techniques for microdermabrasion encompass diamond-tip handpieces, delicately exfoliating dead cells, and crystal microdermabrasion, where fine crystals are emitted to scrub away outer skin layers [28]. This procedure is safe for all skin types and colors, obviating the need for anesthesia or a protracted recovery period [28-32]. Achieving optimal results typically necessitates multiple sessions, with the required number of treatments contingent upon individual needs [28-32].

Microneedling: Microneedling emerges as a minimally invasive cosmetic procedure that uses fine needles to create microchannels in the skin. This process instigates the body’s innate wound healing response, prompting increased collagen and elastin production and yielding skin texture enhancements, scar reduction, and overall skin tone [33-37]. Microneedling applies to various dermatologic concerns, including acne scars, stretch marks, wrinkles, and enlarged pores [34,36,37]. Thanks to local anesthesia, the procedure is generally well tolerated, with minimal discomfort experienced during treatment. Achieving optimal outcomes typically entails multiple sessions with minimal downtime [34,36,37]. While at-home microneedling devices exist, professional-grade devices are deemed more effective and safer for achieving the desired dermal remodeling. Microneedling is generally considered safe for individuals in good general health, although caution is warranted for those with specific skin conditions, active acne, or recent radiation therapy [34,36,37].

Injectable therapies: Injectable therapies, encompassing botulinum toxin and fillers, represent popular modalities for facial rejuvenation in the aging population. To optimize outcomes, it is advisable to allow at least two weeks to elapse post-Botox or filler injections before undergoing additional treatments such as chemical peels or laser resurfacing. Combining injectables with adjunctive treatments like chemical peels, microneedling, laser resurfacing, facials, dermaplaning, and LED light therapy can synergistically enhance results. A healthy lifestyle, including skincare regimens, dietary considerations, regular exercise, and sun protection, is pivotal for maximizing outcomes [38]. Intradermal therapy, commonly called mesotherapy, entails micro-intradermal injections of medications into the skin and holds promise for various clinical conditions within dermatology. Although further clinical studies are warranted to standardize its application in dermatologic pathologies, mesotherapy exhibits potential in pain management and other dermatologic conditions [39]. Soft tissue fillers, notably hyaluronic acid (HA), are frequently employed for skin rejuvenation owing to their minimal allergic risk and reversible effects. HA injections, characterized by their rapid administration and enduring results, offer improvements in skin microrelief, wrinkle severity, hydration, volume, and overall skin quality. Diverse formulations of HA, either standalone or combined with adjunctive molecules, cater to varied patient needs. Incorporating mesotherapy with HA can aid in restoring cutaneous function and ameliorating visible signs of aging [7].

Indications and applications

Cosmetic Dermatology

Cosmetic dermatology encompasses a range of medical procedures and techniques tailored to enhance appearance rather than solely address medical conditions. These interventions are specifically crafted to revitalize the aging face and sustain a youthful aesthetic. Among the most prevalent techniques utilized are chemical peels, intradermal fillers, and botulinum toxin injections, each targeting critical facets of facial aging, such as photodamage, volume depletion, and dynamic wrinkles. Additionally, minimally invasive procedures like microneedling utilize fine needles to create microchannels in the skin, prompting the release of growth factors and stimulating collagen production. Unlike traditional plastic surgery, cosmetic dermatology treatments leverage the body’s innate repair mechanisms to enhance skin health and appearance. They play a vital role in rectifying cumulative skin damage and mitigating natural aging. These treatments form an integral component of a comprehensive skincare regimen, complementing medical interventions prescribed by healthcare professionals [7,40,41].

Treatment of Benign Skin Lesions

Managing benign skin lesions often involves employing a range of minimally invasive dermatosurgical procedures. Laser surgery emerges as a rapid, safe, and efficient method for removing many common benign skin lesions, obviating the need for traditional surgical tools like scalpels or sutures [42]. Additionally, chemical peels, intradermal fillers, botulinum toxin injections, and microneedling represent alternative minimally invasive interventions capable of addressing diverse skin concerns, including benign lesions [7]. The urgency of performing these procedures hinges on factors such as the type of lesion and the patient’s circumstances. For minimally symptomatic or asymptomatic benign lesions such as cysts, lipomas, acrochordons, scars, and keloids, surgical intervention may be deferred until regulatory agencies authorize the resumption of elective surgeries [43].

Management of Precancerous and Cancerous Lesions

Precancerous lesions: Early-stage precancerous lesions can be effectively and swiftly managed using thermal ablation or cryotherapy, ensuring safe treatment outcomes [44]. Additionally, topical medications like 5-fluorouracil, imiquimod, diclofenac, and ingenol mebutate offer alternative avenues for destroying actinic keratoses, a typical precancerous lesion [45]. Treatment of varying grades of cervical intraepithelial neoplasia proves highly efficacious, characterized by simplicity and safety. Comprehensive treatment entails addressing the entire transformation zone of the cervix, employing either ablative techniques such as cryotherapy or thermal ablation. The choice of treatment modality is contingent upon factors such as lesion size, location, and the nature of the transformation zone [46].

Cancerous lesions: The approach to treating skin cancer hinges on several factors, including lesion type, size, and location. Early basal and squamous cell skin cancers can often be managed through cryotherapy, topical medications, or photodynamic therapy, facilitating effective tumor control [45]. In more advanced cases, interventions such as surgical excision, radiation therapy, or chemotherapy may be warranted to address the malignancy comprehensively [45]. Treatment strategies for cervical cancer encompass cryotherapy, thermal ablation, or excisional modalities, guided by considerations like lesion size and location [46]. Specific dermatosurgical procedures generate aerosols, heightening the risk of viral transmission, including viruses like COVID-19. Hence, stringent adherence to appropriate personal protective equipment and safety protocols is imperative during these procedures to mitigate potential risks [43].

Scar Revision and Skin Rejuvenation 

Scar revision and skin rejuvenation are two prevalent dermatosurgical procedures, each serving distinct purposes in enhancing skin aesthetics and function. Scar revision aims to mitigate the appearance of scars stemming from various etiologies such as skin injury, surgical incisions, burns, acne, or other dermatological conditions. Employing an array of techniques, including topical treatments, injectable therapies, surface interventions, skin grafts, and surgical revisions with advanced wound closure methods, scar revision endeavors to ameliorate the cosmetic appearance of scars and restore functionality to the afflicted area [47-50]. While scar revision cannot wholly eradicate scars, it significantly improves their aesthetic presentation. On the other hand, skin rejuvenation procedures aspire to enhance skin aesthetics, address specific skin concerns, and foster skin health. Common modalities encompass chemical peels, intradermal fillers, botulinum toxin injections, microneedling, laser resurfacing, radiofrequency skin tightening, and platelet-rich plasma therapy. These procedures effectively target various issues, including photodamage, volume loss, dynamic wrinkles, scars, acne, melasma, hyperhidrosis, and alopecia, and even facilitate transdermal drug delivery [47,50,51]. While contraindications and limitations vary depending on the procedure, they generally exclude conditions such as active acne, herpetic lesions, moderate to severe chronic skin diseases, blood dyscrasias, and anticoagulant patients. Adverse reactions may occur, although typically minor and transient, particularly with deeper procedures carrying risks such as infection, scarring, and allergic reactions [47-50].

Clinical outcomes and efficacy

Cosmetic Outcomes

In cosmetic dermatology, patient-reported outcome measures (PROMs) play a pivotal role, particularly in elective procedures where patient satisfaction reigns supreme. Despite their significance, the validation and exploration of PROMs in the context of cosmetic procedures still need to be improved. Few PROMs have been specifically designed to gauge patient satisfaction and overall quality, with an even smaller subset addressing critical determinants of quality of life, such as psychosocial impact [52]. Studies conducted in the realm of breast conservation surgery and radiation therapy for multiple ipsilateral breast cancer have unveiled an intriguing trend: patients often rate cosmetic outcomes more positively than clinicians. This underscores the importance of patient self-assessment, as individuals ultimately bear the brunt of treatment outcomes. However, questions linger regarding the reproducibility and susceptibility of patient-reported results to psychosocial influences [53,54]. To comprehensively evaluate cosmetic outcomes, a multifaceted approach encompassing subjective patient assessments, objective measurements, and the grading of skin damage is recommended. Patient self-assessments hold particular significance due to their direct implications for post-treatment quality of life [53,54]. Recognizing the growing importance of understanding patient perspectives, especially in dermatologic surgery and cosmetic dermatology, there is a mounting acknowledgment of the necessity to integrate PROMs into evaluating treatment outcomes. As the healthcare landscape evolves, with increasing demand for cosmetic procedures and rising incidences of skin cancer diagnoses, the incorporation of PROMs stands poised to enrich the assessment of treatment efficacy beyond conventional clinical metrics [52].

Success Rates in Lesion Removal

The efficacy of lesion removal procedures varies depending on the specific technique utilized. Mohs micrographic surgery (MMS) is an exceptionally effective method for treating skin cancer, boasting cure rates of up to 99% for primary skin cancers when skillfully performed by highly trained Mohs surgeons [55]. MMS entails meticulously removing cancerous cells while preserving surrounding healthy tissue, yielding success rates approaching nearly 100% [55]. In contrast, other minimally invasive dermatosurgical procedures such as chemical peels, intradermal fillers, and botulinum toxin injections have shown improved clinical outcomes in approximately two-thirds of clinical trials, with slightly over half of these trials reporting better aesthetic results [7]. However, more extensive randomized trials are warranted to establish these procedures’ efficacy across various applications [7]. Regarding PROMs, using validated PROMs is paramount for assessing patient satisfaction and quality of life in cosmetic dermatology [7]. Nonetheless, the validation and study of PROMs in patients undergoing cosmetic procedures remain limited, with most existing instruments undergoing variable and limited development and validation [7]. In the context of hidradenitis suppurativa, adalimumab, a biologic agent, has demonstrated efficacy and safety when used with surgery for moderate to severe cases. Patients treated with adalimumab exhibited a significantly improved clinical response compared to those receiving a placebo [52].

Long-Term Results and Recurrence Rates

The long-term outcomes and recurrence rates of minimally invasive dermatosurgical procedures exhibit variability contingent upon the specific procedure and treated condition. For instance, a study investigating wide surgical excisions for hidradenitis suppurativa reported a minimal recurrence rate of 2.5% [56]. Similarly, research on deroofing for pilonidal sinus disease indicated a low recurrence rate; however, further investigations are warranted to assess long-term recurrence rates comprehensively [57]. In contrast, a retrospective comparative study comparing surgical excision and repair with nonsurgical and ablative treatments for basal cell carcinoma revealed that cryotherapy yielded recurrence rates ranging from 6.3% to 39% after a two-year follow-up period [58]. PROMs serve as crucial tools for evaluating patient satisfaction and quality of life in the context of cosmetic procedures [52]. Establishing the long-term efficacy and recurrence rates of minimally invasive dermatosurgical procedures across diverse applications necessitates more extensive randomized trials [52]. These studies are essential for providing robust evidence to guide clinical decision-making and optimize patient care in dermatologic surgery.

Comparison with traditional surgical methods

Advantages of Minimally Invasive Techniques

Minimally invasive procedures offer several advantages over traditional surgeries, beginning with smaller incisions that reduce scarring and blood loss during the operation [59]. These smaller incisions contribute to a more aesthetically pleasing outcome and decrease the risk of potential complications associated with larger incisions. Moreover, patients undergoing minimally invasive surgery often experience less post-operative pain and discomfort compared to those subjected to traditional surgical techniques [59]. This reduction in trauma to surrounding tissues translates to diminished tissue damage and nerve irritation, necessitating smaller doses of pain relievers for adequate pain management. In addition to decreased pain, minimally invasive procedures also facilitate faster recovery times due to the reduced trauma inflicted on the body [59-61]. This swifter recovery process allows patients to resume their normal activities sooner, minimizing disruptions to daily life and expediting the return to work or other responsibilities. Furthermore, these procedures are associated with a decreased likelihood of infection as the smaller incisions minimize the exposure of internal tissues to external contaminants, resulting in lower hospital readmission rates [60].

**Figure 1:**
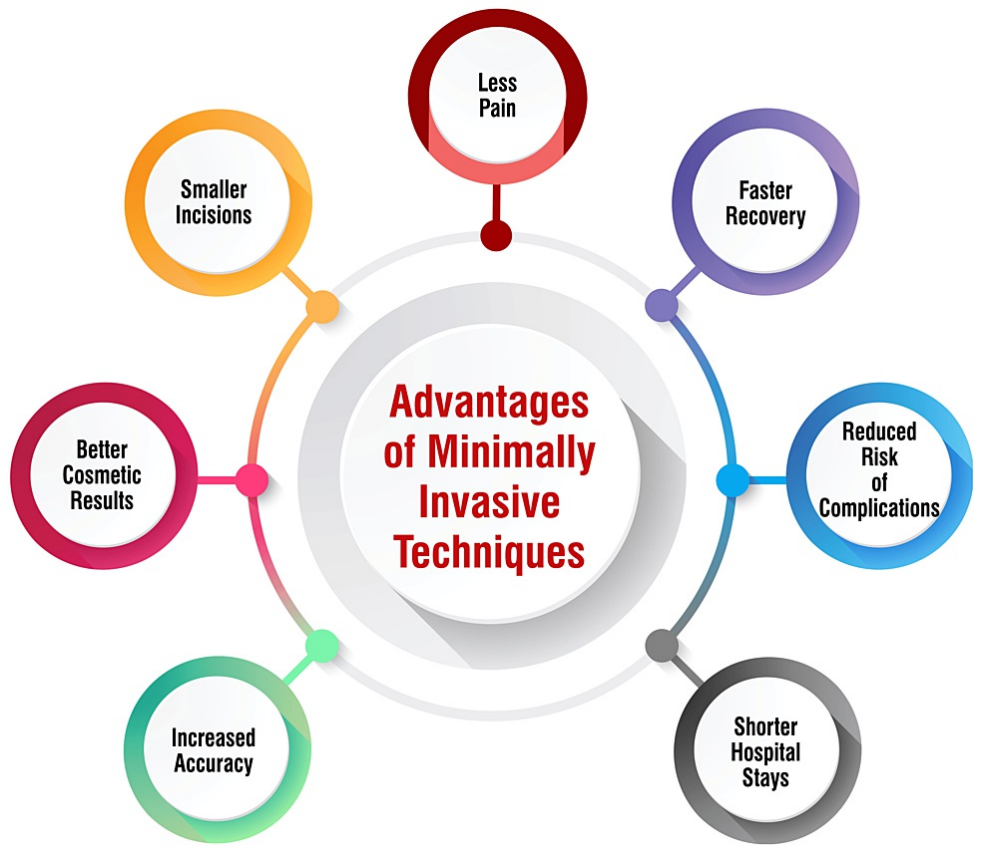
Advantages of minimally invasive techniques Image credit: Soham Meghe

Another notable advantage of minimally invasive techniques is the shorter hospital stays experienced by patients undergoing these procedures [60]. This abbreviated hospitalization period reduces healthcare costs and promotes patient autonomy and comfort by facilitating a quicker transition to home-based recovery. Moreover, minimally invasive surgeries often incorporate video-assisted equipment, providing surgeons with enhanced visualization and magnification of internal organs [60]. This heightened precision enables surgeons to perform procedures more accurately, minimizing the risk of inadvertent tissue damage and optimizing patient outcomes. Additionally, minimally invasive surgeries yield excellent cosmetic results with minimal scarring [59-61]. The inconspicuous scars from these procedures often fade over time, further enhancing their aesthetic appeal for patients. These benefits make minimally invasive techniques an attractive option for patients seeking surgical interventions across various medical specialties, offering superior outcomes, expedited recovery, and diminished post-operative discomfort compared to traditional open surgeries [60]. The advantages of minimally invasive techniques are shown in Figure 1.

Limitations and Challenges

Minimally invasive dermatosurgical procedures, while offering numerous benefits, have limitations and challenges. One prominent concern is ensuring patient safety, as all surgical interventions, including minimally invasive ones, inherently carry risks [62]. Despite efforts to minimize invasiveness, complications can still arise, necessitating meticulous attention to patient selection, preoperative assessment, and intraoperative management to mitigate potential adverse events. Furthermore, an unmet need exists for procedural dermatology, encompassing invasive conventional dermatologic surgeries involving the significant use of knives and sutures and minimally invasive procedures [2]. While minimally invasive techniques have garnered attention for their favorable outcomes, traditional surgical methods may still be indispensable in some instances, highlighting the importance of a comprehensive approach to dermatosurgical practice.

The emergence of the COVID-19 pandemic has introduced additional challenges to dermatosurgical practice, with heightened concerns surrounding patient and healthcare worker safety, risk stratification, and modifications to procedures that generate aerosols [43]. Adhering to stringent infection control measures and adapting protocols to mitigate transmission risks have become imperative in ensuring the continuity of dermatosurgical services amid the ongoing public health crisis. Moreover, while minimally invasive procedures offer considerable advantages, they may not universally apply to all patients or conditions [63]. Certain anatomical or pathological factors may preclude minimally invasive techniques, necessitating a personalized approach to treatment selection based on individual patient characteristics and clinical circumstances. Lastly, while technological innovation lies at the forefront of the new wave of minimally invasive surgery, it also poses challenges in cost, availability, and training [63]. Access to cutting-edge equipment and specialized training programs may be limited, particularly in resource-constrained settings. This underscores the importance of fostering equitable access to advanced technologies and investing in professional development initiatives for healthcare providers.

Future directions and innovations

Emerging Technologies

Technological advancements are rapidly reshaping the landscape of dermatology, offering innovative solutions to improve patient care and treatment outcomes. One such advancement is telemedicine, which enables remote consultations and diagnosis, thereby enhancing patient access to care and convenience [64]. By leveraging telehealth solutions, dermatologists can reach patients in remote or underserved areas, facilitate timely consultations, and streamline the diagnostic process, ultimately improving the overall patient experience and outcomes. In addition to telemedicine, robotics is being increasingly explored to enhance precision during dermatological procedures, such as laser therapy [64]. Robotic systems hold the potential to deliver more accurate treatments by enabling precise targeting of lesions and minimizing damage to surrounding healthy tissue. This advancement in technology has the potential to revolutionize procedural techniques, improving treatment efficacy and patient safety.

Artificial intelligence (AI) tools are also pivotal in dermatological diagnostics, facilitating faster and more accurate assessments and treatment planning [64]. By analyzing vast amounts of clinical data and images, AI algorithms can assist dermatologists in diagnosing skin conditions, predicting disease progression, and optimizing treatment strategies. This integration of AI into clinical practice holds promise for enhancing diagnostic accuracy, reducing diagnostic errors, and optimizing patient outcomes. Furthermore, innovations in three-dimensional printing and artificial skin technologies offer personalized treatment options and skin reconstruction techniques [64]. By enabling the creation of custom-made skin grafts and implants tailored to individual patient needs, these advancements hold immense potential for improving aesthetic outcomes, promoting wound healing, and enhancing patient satisfaction. Emerging therapies utilizing genes, viruses, and cell-based approaches are also transforming the field of dermatology, offering novel treatment modalities for various skin conditions [64]. From gene therapies targeting specific genetic mutations to cell-based regenerative therapies promoting tissue repair and regeneration, these innovative approaches hold promise for addressing previously untreatable skin disorders and advancing personalized medicine in dermatology.

**Figure 2:**
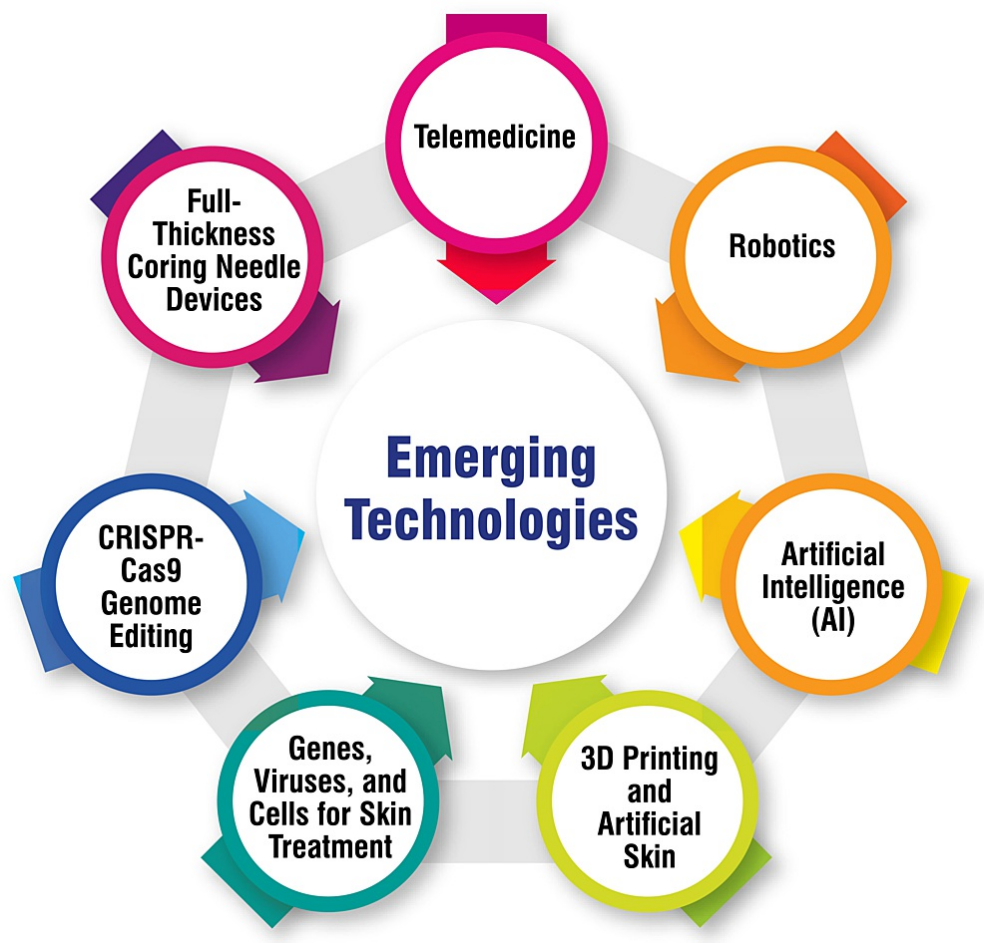
Emerging technologies Image credit: Soham Meghe

Moreover, CRISPR-Cas9 genome editing technology is being explored for genome editing therapies in dermatology, particularly for skin disorders like epidermal blistering [64]. By enabling precise modification of genetic sequences, CRISPR technology offers the potential to correct disease-causing mutations and develop targeted therapies for genetic skin conditions, opening new avenues for treatment and intervention. Additionally, full-thickness coring needle devices are emerging as effective tools for skin rejuvenation by initiating a repair process that forms new collagen and elastin fibers [64]. These devices offer minimally invasive treatment options with minimal scarring, providing patients with safe and effective alternatives to traditional surgical procedures. Collectively, these technological advancements are poised to transform the field of dermatology, ushering in an era of safer, more effective, and personalized care for patients while expanding treatment options for a wide range of skin conditions [64]. By embracing innovation and harnessing the power of technology, dermatologists can revolutionize clinical practice, improve patient outcomes, and advance the field of dermatology into the future. Emerging technologies are shown in Figure 2.

## Conclusions

This review has comprehensively examined minimally invasive dermatosurgical procedures, offering insights into their definition, historical evolution, classification, and applications. We have emphasized the importance of these procedures in modern dermatology, highlighting their relevance in addressing various skin conditions and aesthetic concerns while minimizing patient discomfort and recovery time. By exploring the implications for clinical practice, we underscored the significance of incorporating minimally invasive techniques into dermatologic care, enabling healthcare professionals to deliver personalized and effective treatments to patients. Furthermore, we identified areas for further research, emphasizing the need for ongoing studies to optimize techniques, evaluate long-term outcomes, and explore emerging technologies. Overall, this review underscores the pivotal role of minimally invasive dermatosurgical procedures in shaping the future of dermatologic practice, driving innovation, and ultimately improving patient outcomes.

## Figures and Tables

**Figure 3:**
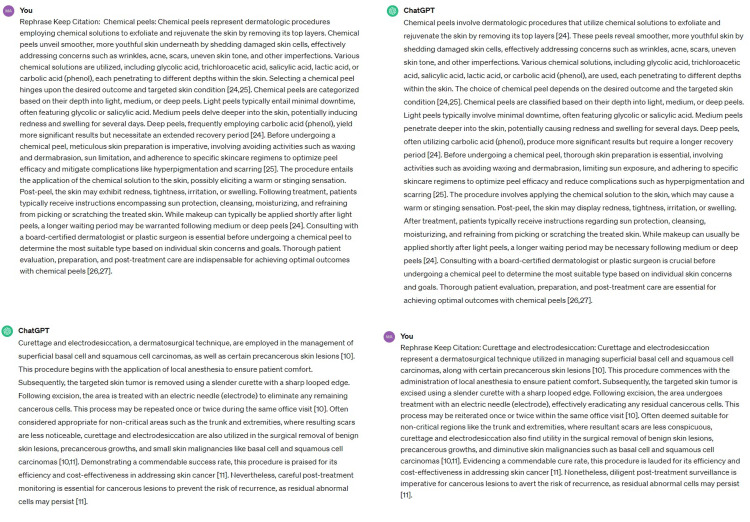
ChatGPT-assisted editing and rephrasing of the review article

## Appendices

The ChatGPT language model (OpenAI, Inc., San Francisco, California, United States) was employed to assist in the formulation of key arguments, structuring the content, and refining the language of our manuscript. It provided valuable insights and suggestions throughout the writing process, enhancing the overall coherence and clarity of the article. It was also utilized to assist in editing and rephrasing the work to ensure coherence and clarity in conveying the findings (Figure 3).
